# Development and validation of a prediction model for premature progesterone elevation during early-follicular phase long-acting GnRH-a long protocol

**DOI:** 10.3389/fendo.2026.1847048

**Published:** 2026-07-22

**Authors:** Tian Ye, Wenqian Fan, Linqing Du, Jing Li, Huijuan Kong

**Affiliations:** 1 Reproductive Medical Center, The First Affiliated Hospital of Zhengzhou University, Zhengzhou, China; 2 Henan Key Laboratory of Reproduction and Genetics, The First Affiliated Hospital of Zhengzhou University, Zhengzhou, China

**Keywords:** *in vitro* fertilization, nomogram, pituitary downregulation treatment, prediction model, premature progesterone elevation

## Abstract

**Background:**

In controlled ovarian stimulation (COS) cycles, premature progesterone elevation (PPE) on the HCG trigger day is associated with adverse pregnancy outcomes after *in vitro* fertilization-embryo transfer (IVF-ET). The aim of this study was to analyze the influencing factors of PPE with the early-follicular phase long-acting GnRH-a long protocol (EFLL) and to explore a dynamic risk-assessment nomogram for PPE to inform risk stratification and early clinical adjustment.

**Methods:**

This was a single-center, retrospective cohort study. Patients who underwent their first IVF cycle at our center from January 2019 to December 2019 were included. All patients were treated with the EFLL protocol. Patients were randomly assigned to the training or validation cohort for nomogram development and testing at a ratio of 8:2. After excluding collinear variables, the remaining candidate predictors were screened by the LASSO regression model, and the nomogram was constructed by multivariate logistic regression analysis. Receiver operating characteristic (ROC) curves and calibration curves with bootstrap were used to evaluate the performance of the nomogram. DCA was used to analyze the clinical efficacy of the model.

**Results:**

A total of 5193 cycles were included—4154 cycles in the training cohort and 1039 cycles in the validation cohort. The incidence of PPE in the training cohort was 20.51%. Multivariate logistic regression showed that BMI, basal FSH, basal progesterone (bP), AMH, AFC, total duration of Gn, total dosage of exogenous HMG, FSH and P on the 6th day of Gn were found to be independent influencing factors of PPE, and a nomogram model was constructed accordingly. The areas under the ROC curves of the training cohort and the validation cohort were 0.683 (95% CI = 0.663-0.702) and 0.622 (95% CI = 0.583-0.662), respectively. The calibration curve showed that the predicted risk of the model was in good agreement with the actual results in both cohorts. Decision curve analysis (DCA) demonstrated the clinical value of this nomogram.

**Conclusions:**

Our nomogram for estimating PPE demonstrates modest discriminative ability and may serve as a preliminary risk stratification tool in COS cycles. It may assist clinicians in identifying patients at higher risk of PPE, thereby informing clinical judgment.

## 1 Introduction

In *in vitro* fertilization/intracytoplasmic sperm injection and embryo transfer (IVF/ICSI-ET), controlled ovarian stimulation (COS) plays a central role. Premature progesterone elevation (PPE) on the HCG trigger day refers to an increase in the progesterone level without the premature surge in the luteinizing hormone (LH) level (1). The probability of PPE in IVF cycle is approximately 5%-38% (2).

Prior studies have indicated that PPE may have a negative impact on the outcome of fresh embryo transfer (3, 4). In 1991, Schoolcraft et al. suggested that PPE was related to a decrease in the clinical pregnancy rate (5). A large-scale meta-analysis of more than 55,000 cycles provided convincing evidence to support the hypothesis that the premature increase in the serum progesterone concentration is related to a low pregnancy rate (6). Previous studies have confirmed that PPE in the late follicular phase is an independent risk factor affecting the clinical pregnancy rate and live birth rate of embryos transferred during fresh IVF cycles (7). Even in populations with good prognoses, the PPE is still negatively correlated with the live birth rate in fresh cycles (1). However, the mechanism of the effect of PPE on IVF outcomes in fresh cycles is still controversial. Although PPE is associated with reduced pregnancy rates in fresh cycles, its negative effects on pregnancy outcomes during frozen-thawed embryo transfer or oocyte donation cycles have not been observed. These findings suggest that PPE may affect pregnancy outcomes during fresh cycles by reducing endometrial receptivity rather than embryo quality (8, 9). However, some authors have proposed that with increasing progesterone levels on the HCG trigger day, the MII oocyte rate, fertilization rate and high-quality embryo rate significantly decrease (10). Therefore, it is speculated that this may further decrease the cumulative pregnancy rate during the subsequent frozen-thawed embryo transfer cycle.

Some authors have proposed adopting a “freeze-all” strategy for patients with PPE on the HCG trigger day. Although this regimen can avoid the negative impact of elevated progesterone level on endometrial receptivity to some extent, it can prolong the time to live birth (TTLB) per oocyte retrieval cycle. For patients using long-acting GnRH-a for pituitary downregulation, this is not the best choice from the perspective of time and economic benefits. In addition, the cryopreservation of embryos may lead to adverse neonatal outcomes, such as an increased incidence of large for gestational age infants (11). Therefore, identifying PPE quickly and accurately is particularly important in clinical practice. Several studies have shown that several factors may be correlated with PPE, including the level of basal progesterone (12), the number of follicles and retrieved oocytes, the daily injected FSH dose, the total FSH dose, the level of estradiol, and whether exogenous LH is added to COS (2). In most of the previous studies, only patients who underwent fresh embryo transfer were included. However, when the progesterone level on the HCG trigger day is above a certain threshold or when the duration of increase is too long, clinicians will choose the “freeze-all” strategy. In other words, patients who did not undergo embryo transfer due to PPE were not included in these studies. However, this group of patients should receive more attention in COS treatment.

Currently, the factors correlating with PPE during COS are not fully understood. There are limited methods available for reducing the occurrence of PPE. There is still a lack of intuitive and individualized clinical models for predicting the risk of PPE. In this article, we retrospectively assessed the common risk factors. To our knowledge, we are the first to develop a dynamic risk-assessment nomogram model by integrating independent factors of PPE through multivariate logistic regression. This model can provide a theoretical basis for clinicians to adjust exogenous Gn individually and may assist in identifying patients at higher risk of PPE.

## 2 Methods

### 2.1 Study population

**Figure 1:**
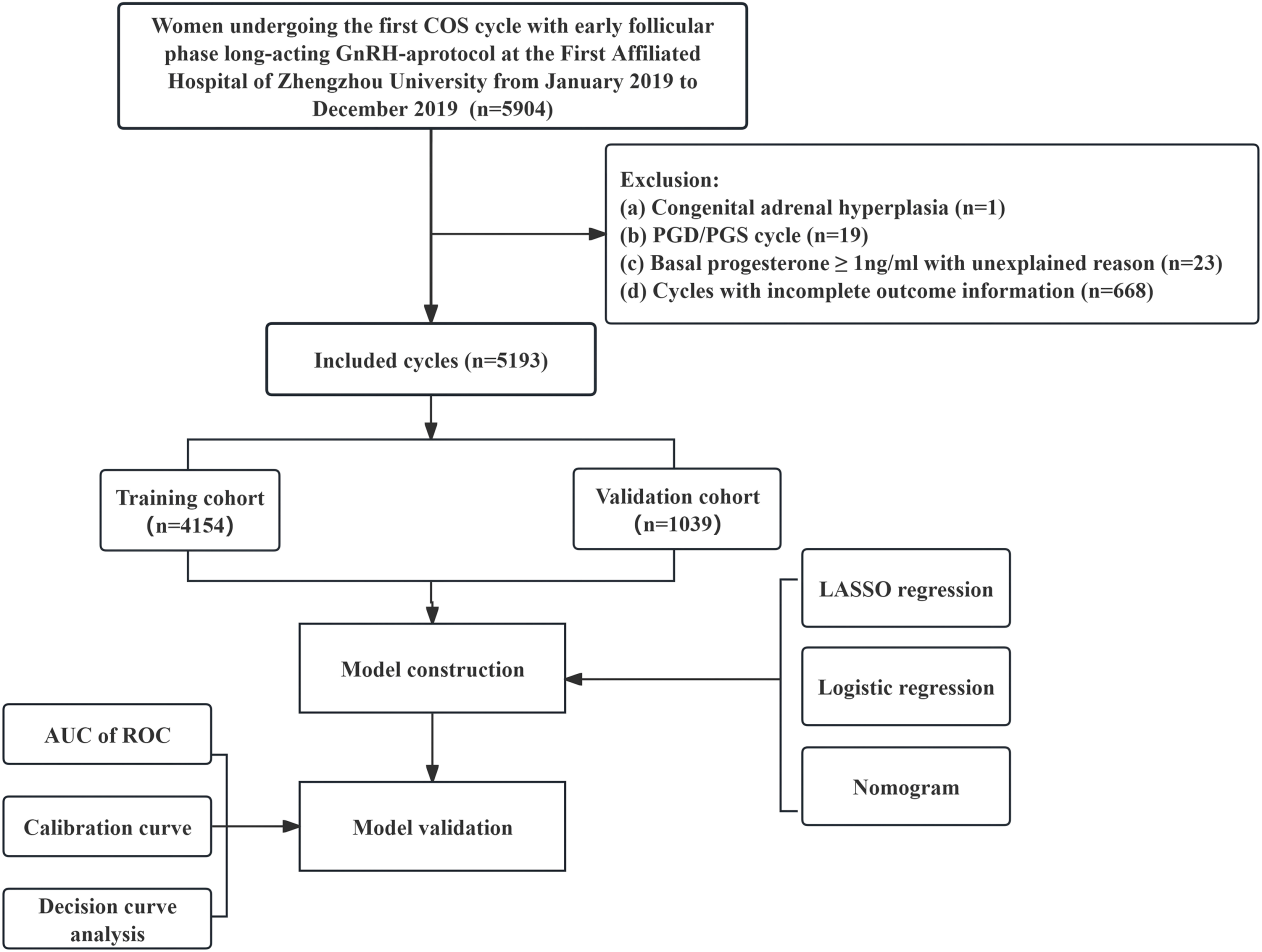
Flowchart of patient screening.

This was a retrospective cohort study of patients receiving assisted reproduction. We included female patients who underwent their first IVF/ICSI cycle at the Reproductive Center of the 1^st^ Affiliated Hospital of Zhengzhou University from January 2019 to December 2019. All patients were treated with the EFLL protocol and completed COS to HCG trigger. The exclusion criteria were as follows: (i) patients with incomplete COS cycles; (ii) patients with congenital adrenal hyperplasia; (iii) patients with unexplained basal progesterone levels ≥ 1 ng/ml; (iv) patients who underwent preimplantation genetic testing (PGT) cycles; (v) patients with incomplete outcome information. Because of the significant decrease in the pregnancy rate and changes in the endometrial gene expression profile, we defined PPE as a progesterone level > 1.5 ng/ml on the HCG trigger day according to previous studies (13, 14). The 668 excluded cycles had all undergone routine serum progesterone testing on the hCG trigger day, but the results were missing in the electronic medical record system due to data retrieval or entry errors. This missingness was not related to any clinical decisions such as early freeze-all for OHSS risk. The flowchart of patient screening is shown in **Figure 1**. This study was approved by the Ethics Committee of the 1^st^ Affiliated Hospital of Zhengzhou University (Approval number: 2024-KY-0038-001). Written informed consent was not required.

### 2.2 Data collection

Clinical data were collected from the Clinical Reproductive Medicine Management System/Electronic Medical Record Cohort Database (CCRM/EMRCD) of the Reproductive Medicine Center of the 1^st^ Affiliated Hospital of Zhengzhou University. The clinical indicators included age, body mass index (BMI), type of infertility, duration of infertility, anti-Müllerian hormone (AMH), basal follicle-stimulating hormone (bFSH), basal luteinizing hormone (bLH), basal estradiol (bE2), basal progesterone (bP) levels, antral follicle count (AFC), and hormone levels (FSH, LH, E2, P) on the 6^th^ day of exogenous Gn, total duration days of exogenous Gn, total dose of exogenous FSH, total dose of exogenous HMG, type of exogenous FSH, and progesterone level on the HCG trigger day.

### 2.3 Controlled ovarian stimulation protocol

The EFLL protocol was performed according to our previous research from our center (15). The patients were given a standard full dose (3.75 mg) of long-acting GnRH agonist (Diphereline, Ipsen, France) for pituitary downregulation on Days 2–4 of the menstrual period. Exogenous Gn was administered for controlled ovarian stimulation when the pituitary downregulation criteria were met (no functional cysts in the ovaries, follicle diameter 3–5 mm, E2 < 30 pg/ml, FSH < 5 mIU/ml, LH < 5 mIU/ml, endometrial thickness < 5 mm). The starting dose of exogenous Gn was based on patient age, BMI, AMH concentration, AFC, and clinician experience. The dose of Gn remained constant for the first 5 days. On the 6th day of exogenous Gn administration, all the patients returned to the hospital for transvaginal ultrasonography, and the serum sex hormone levels were measured. Medication was adjusted according to the development of follicles and changes in hormone levels. HCG was given to trigger ovulation when the leading follicle reached a mean diameter of 20 mm or when at least 3 follicles > 17 mm were present. Transvaginal ultrasound was performed by our experienced physicians in accordance with standardized procedures, and the ultrasound machine used was the same (GE Voluson E10, the United States).

### 2.4 Sex hormone assessment

The levels of AMH, bFSH, bLH, bE2 and bP were determined on Days 2–4 of the menstrual period. FSH, LH, E2 and P were determined on the 6th day of treatment with exogenous Gn, and the P level was determined on the day of hCG induction. A validated electrochemiluminescence immunoassay (Cobas 12145383) was used to detect the hormone. The detection limit and sensitivity of the method were 0.03 ng/ml and 0.15 ng/ml, respectively. The intra-assay and inter-assay coefficients of variation were 3.0 and 5.5%, respectively. The same detection method was utilized throughout the study, and the data were calibrated regularly to reduce unnecessary errors.

### 2.5 Statistical analysis

The classified data were expressed as the frequency and percentage (%), and the median (interquartile distance) was used for continuous data that did not conform to a normal distribution. Overall, patients were randomly divided into a training cohort and a validation cohort at a ratio of 8:2. In the training cohort, the Mann–Whitney U test or χ^2^ test was used for univariate analysis. Variables with a VIF exceeding 5 were excluded, and the remaining non-collinear variables were subsequently included in the least absolute shrinkage and selection operator (LASSO) regression. The covariates with nonzero regression coefficients were screened for further analysis via multivariate logistic regression. A nomogram was constructed based on the independent influencing factors of *p* < 0.05 in the multivariate logistic regression. The receiver operating characteristic (ROC) curve was drawn, and the area under the curve (AUC) was calculated to test the discrimination of the nomogram in the training and validation cohorts. Calibration curves with bootstrap were used to assess the consistency of the actual results with the predicted results. To evaluate the net benefit threshold of the prediction, decision curve analysis (DCA) was conducted. Additionally, a sensitivity analysis was conducted using an alternative PPE threshold of >1.2 ng/ml to test the robustness of our findings. All the statistical analyses were performed with R (version 4.3.2). *P* < 0.05 was considered to indicate statistical significance in general situations. A completed Compliance with Transparent Reporting of a multivariable prediction model for Individual Prognosis or Diagnosis (TRIPOD) checklist is available in **Supplementary Table 1**.

## 3 Results

### 3.1 Demographics and general characteristics

The baseline demographics and general characteristics were summarized in **Table 1**. Our study included a total of 5193 cycles according to the inclusion criteria. There were 4154 cycles in the training cohort and 1039 cycles in the validation cohort. There was no significant difference in baseline demographics between the two cohorts (*p* > 0.05). The incidence of PPE on the day of HCG trigger was 20.51% and 20.40% in the training cohort and validation cohort, respectively.

**Table 1:** Characteristics of the training and validation cohorts.

Characteristics	Training cohort (n = 4154)	Validation cohort (n = 1039)	P-value
Age (year)	30.00 (28.00, 33.00)	30.00 (28.00, 33.00)	0.736
Infertility type (%)			0.304
Primary infertility	2193 (52.79)	530 (51.01)	
Secondary infertility	1961 (47.21)	509 (48.99)	
Infertility duration (year)	3.00 (2.00, 5.00)	3.00 (2.00, 5.00)	0.449
BMI (kg/m2)	22.80(20.70, 25.40)	22.80 (20.70, 25.30)	0.837
Basal E2 (pg/ml)	33.20 (24.42, 43.65)	33.01 (24.93, 44.32)	0.776
Basal LH (mIU/ml)	5.26 (3.88, 7.04)	5.21 (3.85, 7.04)	0.627
Basal FSH (mIU/ml)	6.51 (5.57, 7.81)	6.51 (5.53, 7.80)	0.771
Basal P (ng/ml)	0.25 (0.14, 0.37)	0.25 (0.14, 0.38)	0.881
AMH (ng/ml)	3.16 (1.88, 5.12)	3.14 (1.87, 5.17)	0.884
AFC	16.00 (10.00, 22.00)	16.00 (10.00, 22.00)	0.757
Total dosage of exogenous FSH (IU)	1987.50 (1487.50, 2700.00)	1987.50 (1450.00, 2700.00)	0.734
Type of exogenous FSH used (%)			0.293
Recombinant FSH	3835 (92.32)	949 (91.34)	
Urinary FSH	319 (7.68)	90 (8.66)	
Total duration days of exogenous Gn (day)	13.00 (12.00, 15.00)	13.00 (12.00, 15.00)	0.882
Total dosage of exogenous HMG (IU)	262.50 (150.00, 450.00)	262.50 (150.00, 450.00)	0.795
E2 on the 6th day of exogenous Gn (pg/ml)	84.50 (48.61, 151.00)	91.52 (51.91, 153.30)	0.130
LH on the 6th day of exogenous Gn (mIU/ml)	0.38 (0.22, 0.58)	0.37 (0.20, 0.57)	0.082
FSH on the 6th day of exogenous Gn (mIU/ml)	8.90 (7.16, 12.2)	8.82 (6.97, 12.25)	0.557
P on the 6th day of exogenous Gn (ng/ml)	0.05 (0.05, 0.11)	0.05 (0.05, 0.11)	0.836
Group (%)			0.940
Non-PPE	3302 (79.48)	827 (79.60)	
PPE	852 (20.51)	212 (20.40)	

Variables were presented as median (interquartile range), or n (%).

BMI, body mass index; LH, luteinizing hormone; FSH, follicle-stimulating hormone; P, progesterone; AMH, anti-Müllerian hormone; AFC, antral follicle count; Gn, gonadotropin; HMG, human menopausal Gonadotropin; PPE, premature progesterone elevation.

### 3.2 Baseline characteristics between PPE and non-PPE groups

For the training cohort, **Table 2** displays the baseline characteristics grouped as those with or without PPE. In the training cohort, 852 cycles were included in the PPE group, and 3302 cycles were included in the non-PPE group. There were significant differences in age, bE2, bP, and AMH levels; and FSH and P levels on the 6^th^ day of treatment with exogenous Gn between the two groups (*p* < 0.05; **Table 2**).

**Table 2:** Univariate analysis of influencing factors of PPE in training cohort.

Characteristics	Non-PPE (n = 3302)	PPE (n = 852)	P-value
Age (year)	30.00 (28.00, 33.00)	30.00 (28.00, 34.00)	0.013
Infertility type (%)			0.712
Primary infertility	1748 (52.94)	445 (52.23)	
Secondary infertility	1554 (47.06)	407 (47.77)	
Infertility duration (year)	3.00 (2.00, 5.00)	3.00 (2.00, 5.00)	0.677
BMI (kg/m2)	23.00 (20.80, 25.50)	22.00 (20.20, 24.30)	< 0.001
Basal E2 (pg/ml)	32.63 (24.01, 43.17)	34.70 (26.00, 46.17)	< 0.001
Basal LH (mIU/ml)	5.25 (3.85, 7.04)	5.29 (3.99, 6.98)	0.616
Basal FSH (mIU/ml)	6.49 (5.54, 7.82)	6.55 (5.67, 7.74)	0.604
Basal P (ng/ml)	0.24 (0.14, 0.36)	0.28 (0.17, 0.40)	< 0.001
AMH (ng/ml)	3.26 (1.91, 5.38)	2.88 (1.80, 4.27)	< 0.001
AFC	16.00 (10.00, 22.00)	14.00 (10.00, 19.00)	< 0.001
Type of exogenous FSH used			0.726
Recombinant FSH	3046 (92.25)	789 (92.61)	
Urinary FSH	256 (7.75)	63 (7.39)	
Total dosage of exogenous FSH (IU)	19132.50(1425.00, 2687.50)	2225.00 (1725.00, 2775.00)	< 0.001
Total duration days of exogenous Gn (day)	13.00 (12.00, 15.00)	14.00 (13.00, 15.00)	< 0.001
Total dosage of exogenous HMG (IU)	262.50 (150.00, 450.00)	262.50 (150.00, 450.00)	0.286
E2 on the 6th day of exogenous Gn (pg/ml)	82.87 (46.62, 152.80)	87.56 (54.18, 143.90)	0.134
LH on the 6th day of exogenous Gn (mIU/ml)	0.39 (0.23, 0.58)	0.37 (0.22, 0.59)	0.365
FSH on the 6th day of exogenous Gn (mIU/ml)	8.67 (7.02, 11.70)	9.93 (7.86, 13.54)	< 0.001
P on the 6th day of exogenous Gn (ng/ml)	0.05 (0.05, 0.10)	0.05 (0.05, 0.13)	< 0.001

Variables were presented as median (interquartile range), or n(%).

BMI, body mass index; LH, luteinizing hormone; FSH, follicle-stimulating hormone; P, progesterone; AMH, anti-Müllerian hormone; AFC, antral follicle count; Gn, gonadotropin; HMG, human menopausal Gonadotropin; PPE, premature progesterone elevation.

### 3.3 Preliminary screening of PPE predictors

**Figure 2:**
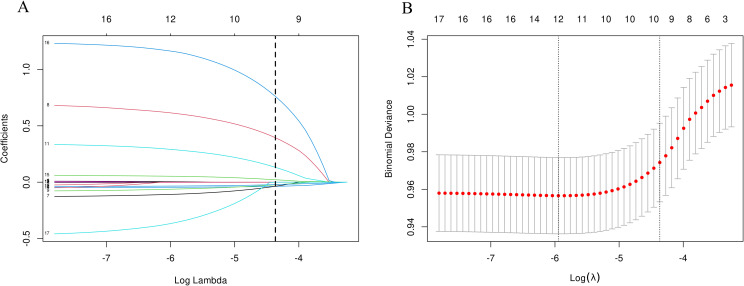
Best match factor screening by lasso regression. **(A)** is the Lasso regression path diagram. **(B)** is the plot of the best matching factors screened by the 10-fold cross validation method, and the best matching factors were selected using lambda 1se as the criterion.

We evaluated multicollinearity and excluded the variable with a VIF > 5 (total dose of exogenous FSH). LASSO regression with ten-fold cross-validation and lambda 1se as the criterion of influencing factors. According to the coefficient track diagram, the model screened out 9 variables with nonzero regression coefficients. The best matching predictors were as follows: BMI; bFSH, bP, AMH, and AFC; total dose of exogenous HMG; total duration days of exogenous Gn; the level of FSH and P on the 6^th^ day of exogenous Gn. The screening procedure for LASSO regression was shown in **Figure 2**.

### 3.4 Multivariate logistic regression analysis of PPE

**Figure 3:**
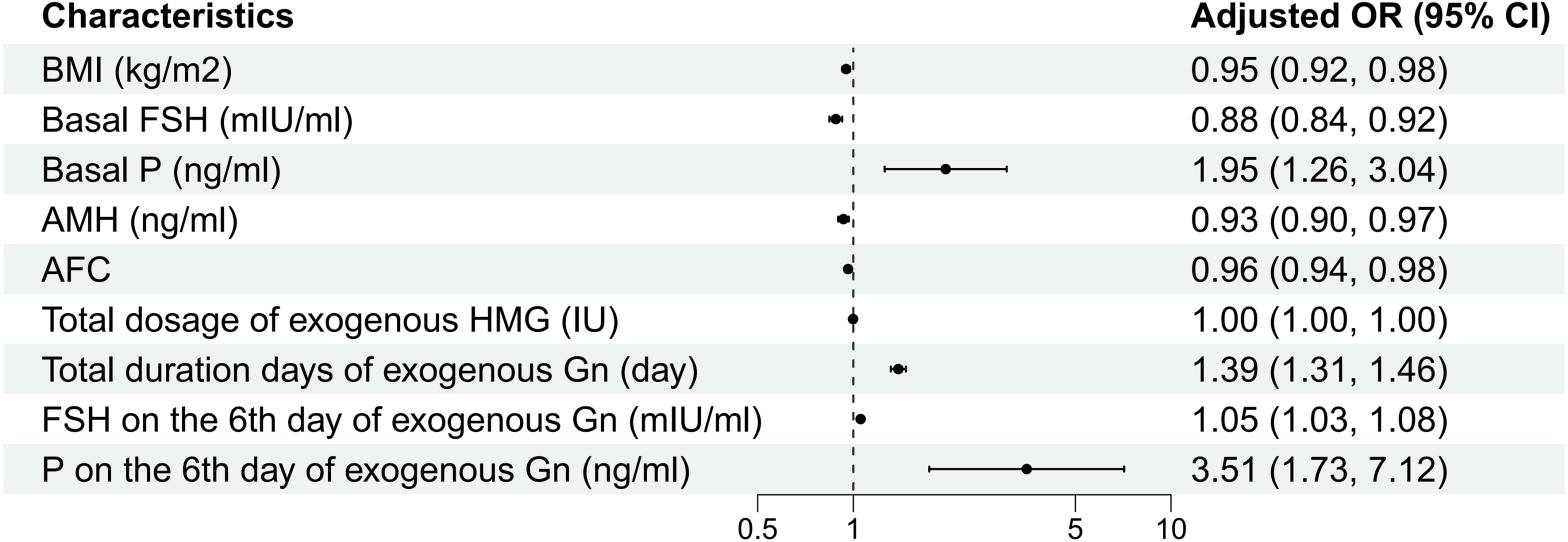
Forest plots of independent influencing factors for PPE by multivariate analysis. BMI, body mass index; P, progesterone; AMH, anti-Müllerian hormone; AFC, antral follicle count; FSH, follicle-stimulating hormone; Gn, gonadotropin.

The 9 variables mentioned above were further included in a multivariate logistic regression analysis. We found that BMI (OR = 0.95, 95% CI = 0.92-0.98, *p* < 0.001), bFSH (OR = 0.88, 95% CI = 0.84-0.92, *p* < 0.001), bP (OR = 1.95, 95% CI = 1.26-3.04, *p* = 0.003), AMH (OR = 0.93, 95% CI = 0.90-0.97, *p* < 0.001), AFC (OR = 0.96, 95% CI = 0.94-0.98, *p* < 0.001), total dose of exogenous HMG (OR = 1.00, 95% CI = 1.00-1.00, *p* < 0.001), total duration days of exogenous Gn (OR = 1.39, 95% CI = 1.31-1.46, *p* < 0.001), FSH on the 6^th^ day of exogenous Gn (OR = 1.05, 95% CI = 1.03-1.08, *p* < 0.001) and P on the 6^th^ day of exogenous Gn (OR = 3.51, 95% CI = 1.73-7.12, *p* < 0.001) were independent factors influencing the level of P on the HCG trigger day (**Figure 3**). Among these factors, BMI, bFSH, AMH and AFC were determined to be independent protective factors for PPE. All VIF values for the nine independent predictors in the final multivariate logistic regression model were below 5, confirming the absence of meaningful multicollinearity.

### 3.5 Construction and validation of a nomogram model

**Figure 4:**
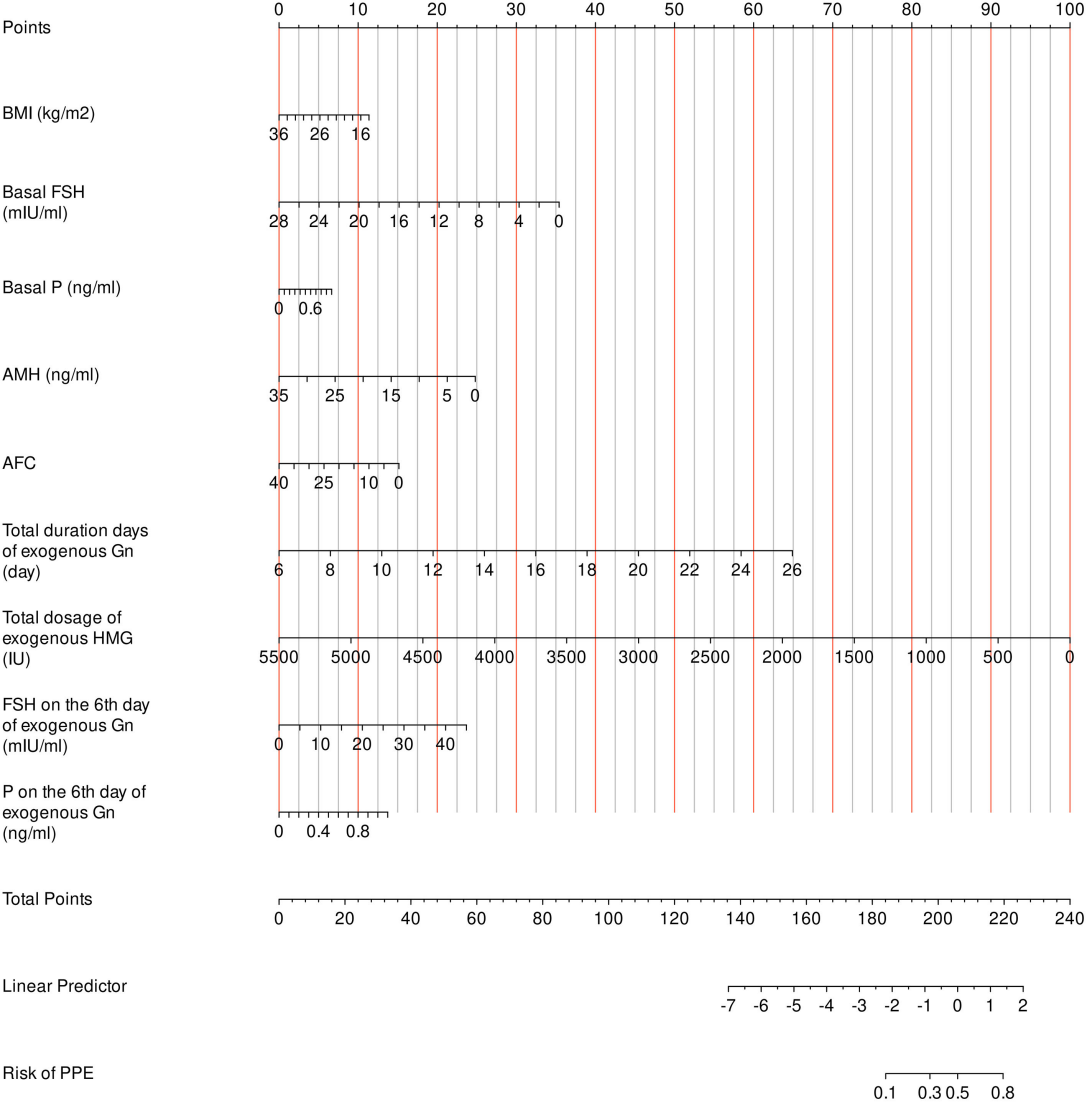
Nomogram of the prediction model for PPE with EFLL. BMI, body mass index; P, progesterone; AFC, antral follicle count; FSH, follicle-stimulating hormone; Gn, gonadotropin; PPE, premature progesterone elevation; EFLL, early-follicular phase long-acting GnRH-a long protocol.

According to the findings of multivariate logistic regression, the logistic regression equation was as follows: log(Y) = -3.161-0.052 × BMI - 0.127× bFSH + 0.669 × bP - 0.071× AMH - 0.038 × AFC + 0.327 × Total duration days of exogenous Gn - 0.002 × Total dosage of exogenous HMG + 0.053 × FSH on the 6^th^ day of treatment + 1.257 × P on the 6^th^ day of treatment. A nomogram that integrates all significant independent factors for PPE on the HCG trigger day is shown in **Figure 4**. The top line of the nomogram corresponds to the score for each factor. Scores for each of these parameters were pooled, with higher scores indicating a greater risk of developing PPE.

**Figure 5:**
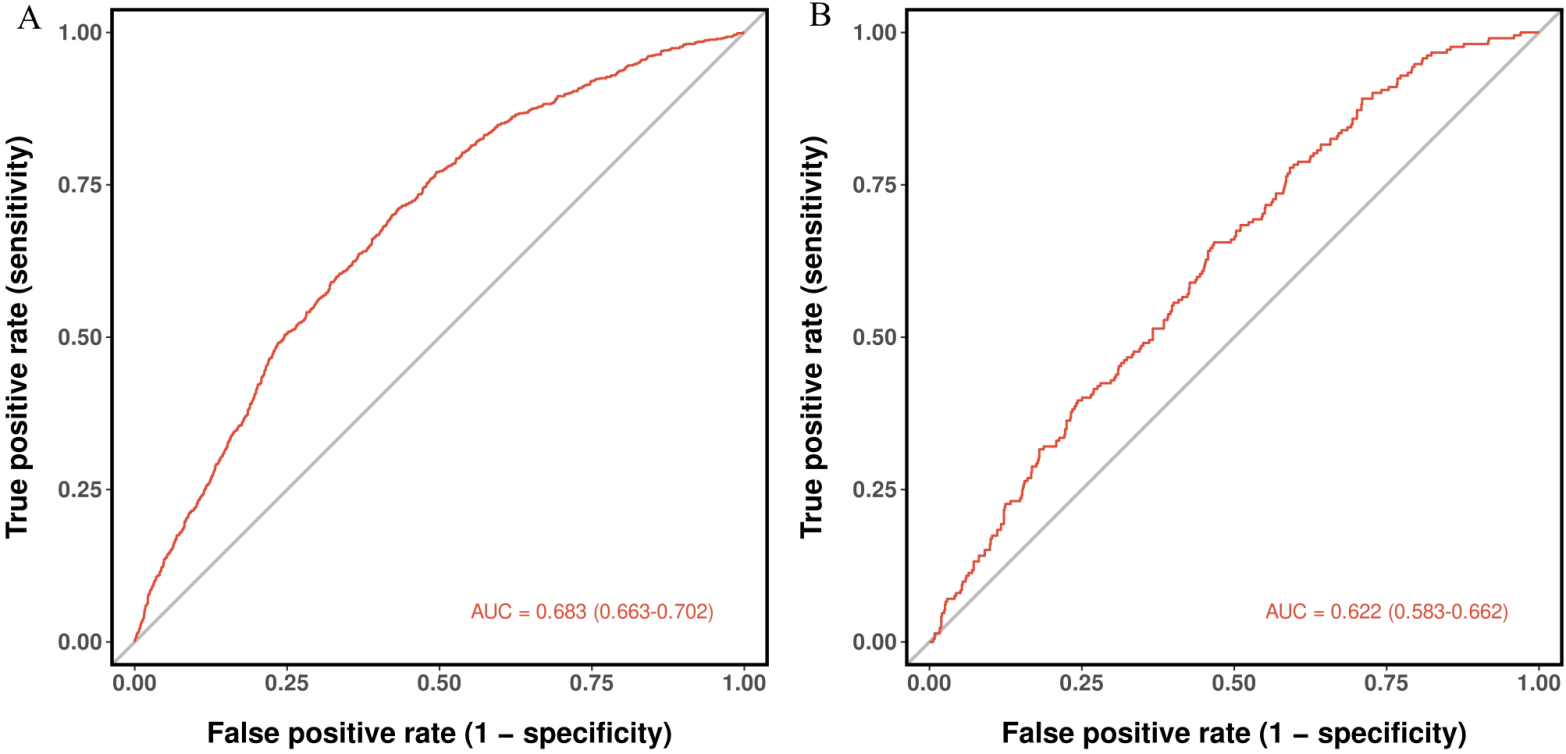
Discriminative power of the nomogram for PPE with EFLL. **(A)** shows the receiver operating curve of the training cohort. The area under the curve was 0.683 (95% CI = 0.663-0.702). **(B)** shows the receiver operating curve of the validation cohort. The area under the curve was 0.622 (95% CI = 0.583-0.662).

**Figure 6:**
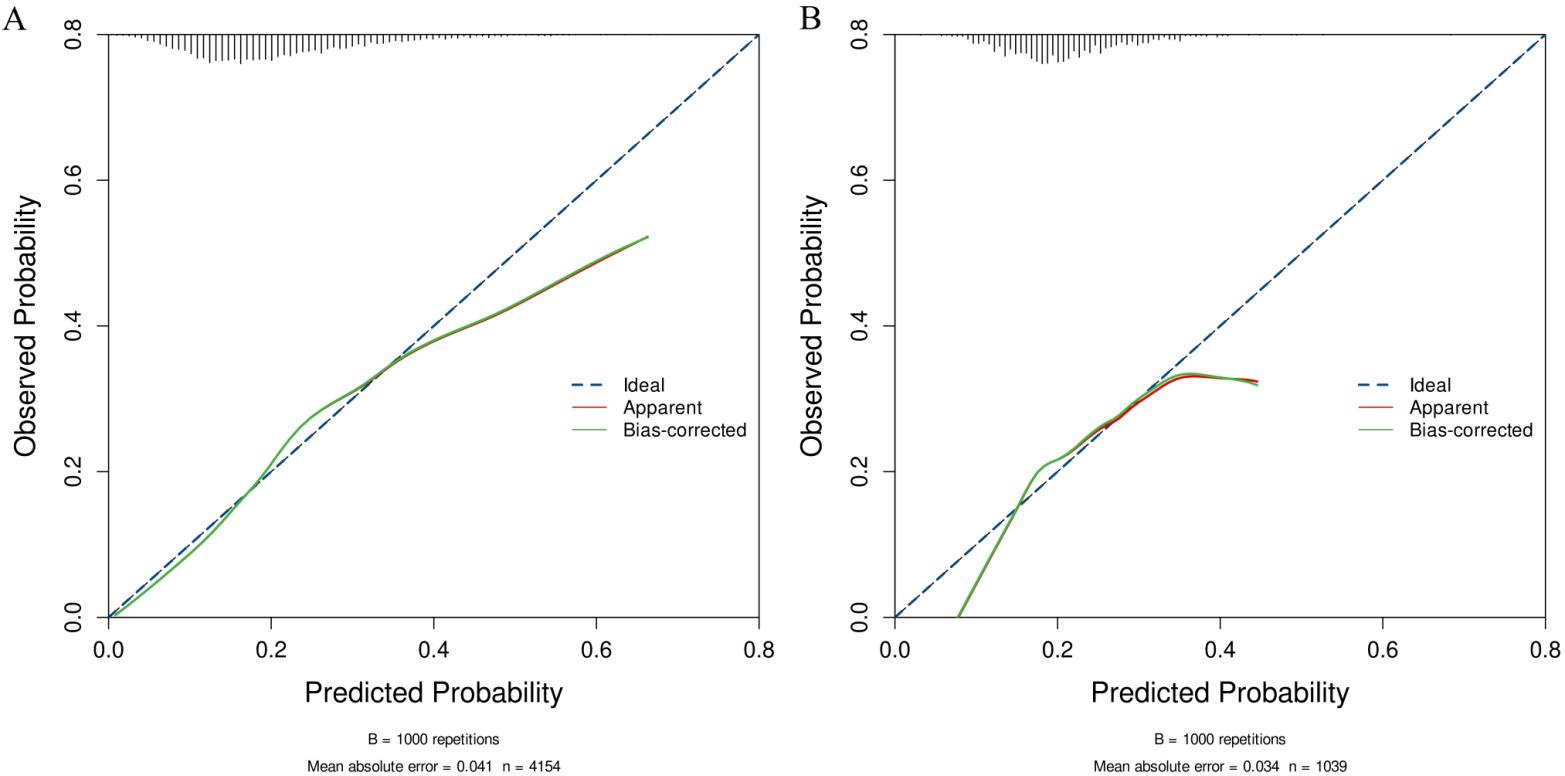
Calibration curve of the nomogram for PPE with EFLL. **(A)** and **(B)** are calibration curves of the training cohort and the validation cohort, respectively.

**Figure 7:**
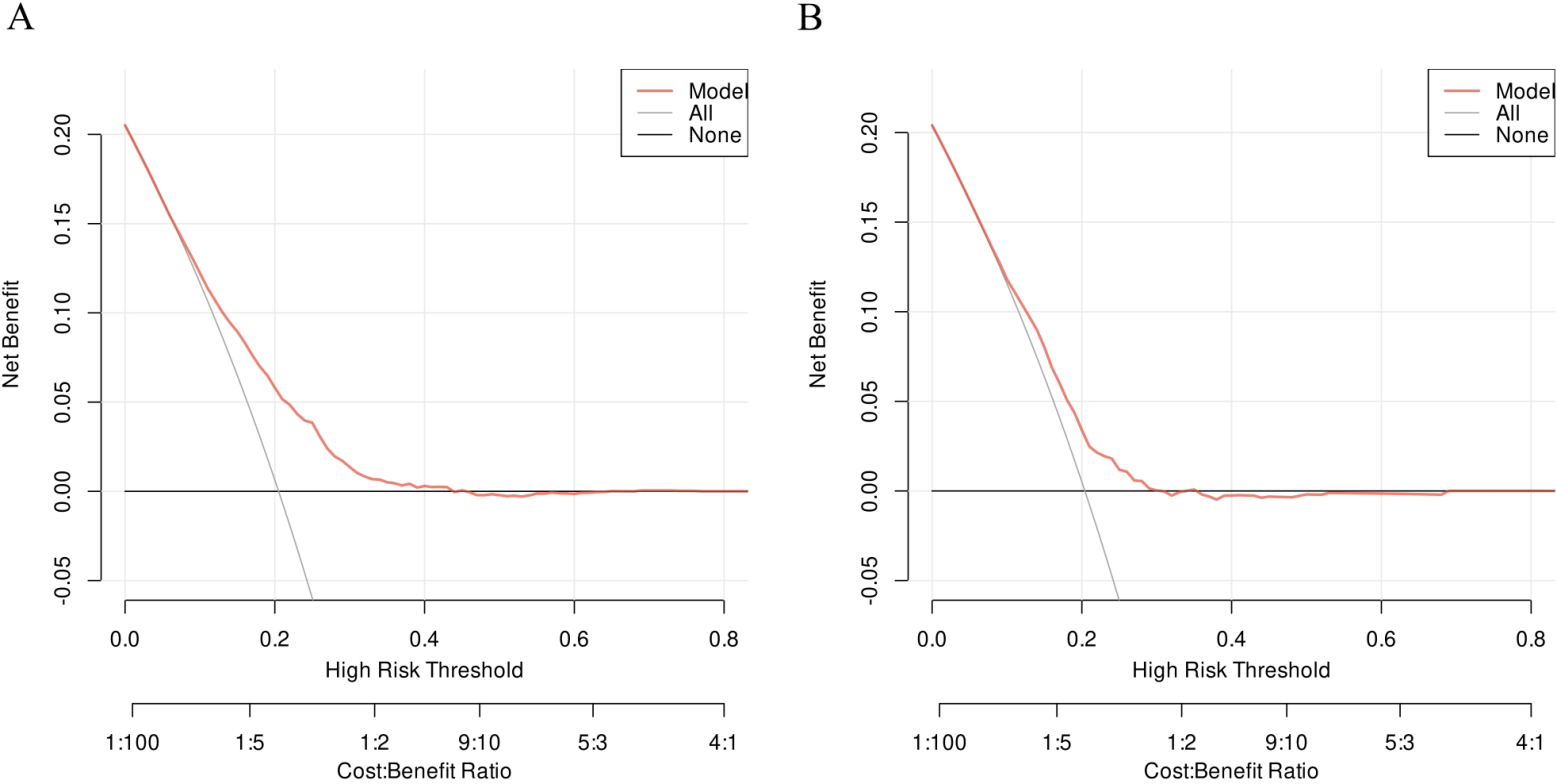
Decision curve analysis of the nomogram for PPE with EFLL. **(A)** and **(B)** are decision curves of the training cohort and the validation cohort, respectively.

In the training cohort, the area under the ROC curve (AUC) was 0.683 (95% CI = 0.663-0.702) with 70.89% sensitivity and 56.84% specificity. A similar result was observed in the subsequent validation cohort. The nomogram displays an AUC of 0.622 (95% CI = 0.583-0.662) with 59.91% sensitivity and 55.99% specificity, suggesting the moderate discriminative ability of the prediction model (**Figure 5**). At the optimal cutoff value of 0.19 determined by the Youden index, the diagnostic performance was further characterized by an accuracy of 59.73% (95% CI: 58.22%–61.22%), a PPV of 29.77% (95% CI: 27.78%–31.76%), and an NPV of 88.33% (95% CI: 86.96%–89.69%) in the training cohort. In the validation cohort, the corresponding values were an accuracy of 56.79% (95% CI: 53.71%–59.82%), a PPV of 25.87% (95% CI: 21.99%–29.74%), and an NPV of 84.49% (95% CI: 81.46%–87.52%). The calibration curves demonstrated good agreement at low to moderate predicted probabilities in both cohorts (**Figure 6**). In the training cohort (panel A), the bias-corrected line tracked closely with the ideal line across most of the range, with only mild departure at the upper tail. However, in the validation cohort (panel B), the bias-corrected line diverged notably from the ideal line at predicted probabilities exceeding approximately 0.35 - 0.40, indicating some overestimation of PPE risk in this higher range. The Hosmer - Lemeshow test showed no significant overall deviation in either cohort (training: χ² = 7.394, p = 0.495; validation: χ² = 8.013, p = 0.432), yet as a global test it may be less sensitive to localized miscalibration at the tails. Given that the majority of patients in our cohort were concentrated at low to moderate predicted probabilities, this departure at higher risk levels is unlikely to substantially alter overall clinical decision-making. Decision curve analysis demonstrated that the nomogram provided net clinical benefit over both the treat-all and treat-none strategies within a threshold probability range of approximately 5% to 30% in the training cohort (**Figure 7A**). Notably, at threshold probabilities exceeding approximately 30%, the net benefit curve dropped below the treat-none line, suggesting limited utility when considering aggressive early intervention exclusively for patients at very high predicted probabilities. In the validation cohort, the range of net benefit was narrower, with the model demonstrating clinical utility primarily within a threshold probability range of approximately 10% to 25% (**Figure 7B**). Given that the observed PPE prevalence in our cohort was approximately 20%, these findings indicate that the nomogram may be clinically useful for guiding early risk stratification and individualized gonadotropin adjustment in patients with moderate-to-high PPE risk, while acknowledging that its utility diminishes at extremely high predicted probabilities.

In a sensitivity analysis using a PPE threshold of >1.2 ng/ml, the core independent predictors and their effect directions remained consistent with the primary analysis (**Supplementary Table 2**), supporting the overall robustness of the model.

## 4 Discussion

Elevated progesterone levels in the late follicular phase during COS may reduce endometrial receptivity and impair embryo quality, possibly prolonging TTLB. Hence, exploring the independent influencing factors of PPE and establishing a prediction model are highly clinically important. In this study, through multivariate regression, we successfully developed a quantifiable and simple-to-use nomogram for estimating PPE on the HCG trigger day during early-follicular phase long-acting GnRH-a long protocol.

Patients with adrenal hyperplasia and unexplained basal progesterone levels > 1 ng/ml were excluded from our study. The ability of the basal progesterone concentration to predict PPE efficacy was maintained even after long-acting GnRH-a pituitary downregulation. In other words, bP was an independent risk factor for PPE. A previous study revealed that bP could predict the occurrence of P > 1.35 ng/ml on the HCG trigger day, even after controlling for confounding factors such as total Gn duration, total FSH dose, and E2 level on the HCG day (12). Both the GnRH-a and GnRH-ant protocols were included in that study. In a study of the GnRH-ant protocol, the authors also found similar results (3). Mutlu et al. reported that a bP > 0.65 ng/ml in GnRH-ant protocol was correlated with a P > 1.5 ng/ml on the HCG trigger day (16). In line with the above previous studies, we confirmed these findings in a much larger sample size (n = 4154 cycles); hence, we can increase the statistical power of the study. Currently, the mechanism through which bP affects PPE is unclear. De Geyter et al. showed that the serum progesterone concentration in the early follicular stage mainly originates from the adrenal gland, whereas in the late follicular stage, it mainly originates from the ovaries (17). Therefore, it is speculated that the progesterone secreted by the adrenal glands in the early follicular phase may persist throughout the whole follicular cycle. The interaction between the ovary and adrenal gland with respect to progesterone secretion still needs to be further explored.

Previous studies have shown that the serum progesterone concentration in the late follicular phase is positively correlated with the number of mature follicles and the serum E2 concentration (6, 18). Unexpectedly, we found that the AFC and AMH concentration were protective factors against PPE. Shahryar K. Kavoussi et al. confirmed that the AMH level was not an independent predictor of PPE (19). These authors suggested that the mechanism of action of PPE during COS treatment may not be simply due to the increase in the number of granulosa cells. Moreover, for POSEIDON’s Groups 1 and 2 patients, for example, the ovarian reserve is not equal to the ovarian response to exogenous Gn (20). For patients with ovarian hyper response, to reduce the risk of ovarian hyperstimulation syndrome, an ovulation trigger combined with *in vitro* maturation may be administered before the follicle diameter reaches the established criterion (21, 22). Thus, the progesterone level on the HCG day could be within the normal range or only slightly elevated. On the other hand, previous research has confirmed that AMH not only inhibits the growth of primordial follicles but also inhibits the sensitivity of ovaries to exogenous FSH stimulation in a mouse model (23). Consequently, the greater the AFC and the greater the AMH level are, the less sensitive the ovaries may be to exogenous FSH stimulation. All of these findings may explain why the AFC and AMH are negatively correlated with PPE.

Our results showed that BMI was an independent protective factor against PPE. These findings are consistent with those of earlier studies indicating that progesterone levels are negatively regulated by BMI (24). With increasing BMI during COS treatment, the risk of follicular hypoplasia increases, and patients need a higher dose and/or longer duration of exogenous Gn stimulation (25, 26). This difference may be related to the downregulation of FSH receptor expression in ovarian granulosa cells in these patients to reduce sensitivity to exogenous Gn stimulation (27). In addition, obesity may also hinder the formation of meiotic spindles in oocytes (28), thus reducing follicular growth and development (29). Therefore, we speculated that BMI may affect the recruitment and development of follicles, ultimately affecting the synthesis of progesterone in the late follicular phase.

In addition to the clinical baseline characteristics of patients, several therapeutic indicators during COS treatment are also correlated with PPE. Our results indicated that the total duration of exogenous Gn were independent risk factors for PPE. These results are consistent with previous studies (30–32). Bosch et al. reported that more stimulation days existed in the group of progesterone level on the HCG trigger day ≥ 3.82 nmol/L. Their results also showed that for every 75 IU increase in the total dose of exogenous FSH, the probability of PPE increased by 1.44-fold (30). Oktem et al. and Anshu Yadav et al. also proposed that longer stimulation days and a greater total dose of exogenous FSH were associated with higher progesterone levels on the HCG trigger day (31, 32). Exogenous FSH is thought to affect serum progesterone secretion through multiple mechanisms: (i) it can stimulate follicular growth by binding to the FSH receptor in granulosa cells; at the same time, (ii) it can directly stimulate the expression of Star protein, cholesterol lyase and 3β-hydroxydehydrogenase (3β-HSD) in granulosa cells causing an increase of progesterone synthesis (33); apart from these, (iii) it can also induce granulosa cells to express a large number of LH receptors, making the follicles hypersensitive to LH-like activity and resulting in increased progesterone levels (34). These results suggest that clinicians should pay more attention to the total duration of exogenous Gn.

Our results also demonstrated that, in the PPE group, the FSH level on the 6^th^ day of treatment with exogenous Gn was significantly greater than that in the non-PPE group (**Table 2**), suggesting that patients in the PPE group received greater exogenous FSH stimulation and more progesterone synthesis on the 6^th^ day of treatment with exogenous Gn. Even after the confounding factors were excluded by multivariate logistic regression, P on the 6^th^ day of treatment with exogenous Gn was still a risk factor for PPE (**Figure 3**). Therefore, clinicians can judge whether exogenous FSH is excessive on the 6^th^ day of Gn treatment in the early stage of COS to adjust the Gn dosage in a timely manner to avoid PPE. The reason for this difference may be that the blood concentration reaches a steady state after repeated administration of exogenous FSH for 5–7 days (35), and the serum FSH concentration at this time can accurately reflect the intensity of FSH stimulation in the ovary (36). D. Kyrou et al. demonstrated that the P levels on the 6^th^ and 8^th^ days of Gn treatment were both correlated with the P level on the HCG day (*r* = 0.321 and *r* = 0.486, respectively; *p* < 0.001 for both correlations) (37). Although the correlation coefficient of the P level on the 8^th^ day of Gn treatment was greater than that on the 6^th^ day, we hope that PPE can be predicted earlier during COS to gain more time for further Gn adjustment to prevent PPE. Consequently, the P level on the 8^th^ day of Gn treatment was not included in our study.

To translate these findings into clinical context, we propose the following framework for interpreting this nomogram. Because total stimulation duration and total HMG dose are only available upon cycle completion, the nomogram in its current form is best understood as a retrospective risk-profiling tool rather than a purely prospective prediction instrument. Its primary utility lies in identifying independent contributors to PPE and quantifying their relative importance once the full stimulation course is known. That said, several variables in the model—particularly BMI, AMH, AFC, and Day 6 FSH and progesterone—are available before or during early stimulation. Among these, Day 6 progesterone and FSH levels emerge as the most actionable early dynamic signals. When these mid-stimulation values are elevated, clinicians may consider reducing the daily FSH dose, adding LH/hMG more cautiously, or shortening the stimulation duration if follicular maturity permits. This proactive approach has two potential advantages: by mitigating excessive steroidogenic stimulation, it may inform the consideration of fresh embryo transfer strategy, though we emphasize that any impact on pregnancy or live birth outcomes was not evaluated in this study; even when PPE ultimately occurs, earlier recognition of risk allows clinicians to counsel patients and adjust expectations earlier in the cycle.

Most of the previous studies on PPE have involved correlation analyses, systematic review and retrospective cohort studies (3, 4, 6, 30). The cutoff value of a single variable was given (12, 16), and the prediction value was low. In our present study, the clinical baseline characteristics of patients and COS-related therapeutic indicators were comprehensively considered. Through multivariate regression analysis, we estimated PPE based on 9 independent factors: BMI, bFSH, bP, AMH, AFC, total duration of Gn, total dosage of exogenous HMG, FSH and P on the 6th day.

It is important to clarify the conceptual nature of this nomogram. Because the model incorporates both pre-treatment baseline variables (BMI, AMH, AFC, bFSH, bP) and dynamic on-treatment measurements (FSH and P on Day 6, total Gn duration, total HMG dose), it should be understood as a dynamic risk-assessment tool rather than a pure baseline prediction model. The Day 6 hormonal markers capture the evolving ovarian response to exogenous stimulation, enabling risk re-stratification at a mid-stimulation timepoint, while the cumulative exposure variables characterize the overall pharmacological milieu. Consequently, this nomogram does not predict PPE before treatment initiation; rather, it refines risk estimation as the cycle unfolds, with the Day 6 parameters serving as early dynamic signals for potential clinical adjustment. This integrative approach incorporates both baseline patient characteristics and dynamic stimulation parameters, offering a more comprehensive risk assessment than single-variable cut-offs. While basal progesterone and Day 6 progesterone have each been associated with PPE risk, they represent single-domain indicators. Our nomogram integrates these with additional independent factors, offering a multidimensional risk assessment. We acknowledge that a direct comparison of predictive performance against simpler models was not performed in this study and should be addressed in future validation. The nomogram provides a visual tool for estimating individualized PPE risk. The AUCs of our nomogram for PPE in training cohort and validation cohort were 0.683 and 0.622, respectively, suggesting a certain degree of discrimination.

Several factors explain the modest discriminative ability. PPE involves complex endocrine biology—granulosa cell steroidogenesis, adrenal contributions, and dynamic ovarian responses—that routine clinical measures simply miss. Our single-center EFLL cohort also lacked the heterogeneity needed to push discrimination higher, and the 20% PPE prevalence naturally caps the positive predictive value. Given the modest AUC of 0.622 in the validation cohort, we caution that this nomogram should be regarded as a supplementary risk-awareness tool rather than a definitive clinical decision instrument.

As we all know, antagonist protocol has gained international preference for its flexibility in recent years, but EFLL remains widely used in China with better pregnancy outcomes (38, 39). We built this nomogram for estimating PPE risk in first-time EFLL cycles. It is a practical tool for this specific setting.

Our study has several limitations that should be acknowledged. First and foremost, the absence of external validation represents the most critical limitation of this study. The validation cohort was generated by random split from the same single-center dataset covering the same time period and the same patient population. While this internal validation approach, supplemented by bootstrap resampling, can assess the model’s stability within this specific cohort, it cannot verify whether the model remains valid when applied to patients from different centers, geographic regions, or clinical settings with varying laboratory assays, stimulation protocols, or physician practices. This severely constrains the generalizability of our findings. Second, we chose P ≥ 1.5 ng/ml as the threshold for PPE because this value was commonly used in the previous literature. However, progesterone assays vary across reproductive centers in terms of detection platforms (e.g., electrochemiluminescence immunoassay, chemiluminescence immunoassay), antibody specificity, and calibration standards, which may introduce systematic bias in absolute progesterone values. The threshold of >1.5 ng/ml in our study was established using the Cobas electrochemiluminescence immunoassay system with regular calibration. Therefore, external application of this nomogram requires careful validation and potential recalibration of the threshold according to local laboratory characteristics. Third, although the 668 excluded cycles with missing hCG-day progesterone values were attributable to electronic medical record retrieval errors rather than clinical decisions, the exclusion of 11.2% of the initial population carries a theoretical risk of selection bias if the missingness was non-random with respect to patient characteristics. Moreover, the AUC of the nomogram we developed in this study was less than 0.7, suggesting moderate prediction efficiency. Because progesterone secretion during ovarian stimulation is governed by complex and finely regulated biological and endocrine mechanisms. In the future research, we look forward to incorporating additional biological markers to achieve a more comprehensive prediction of PPE. Nevertheless, this was a real-world retrospective study based on clinical medical records with a large sample size and strict inclusion and exclusion criteria. The exploration of the PPE prediction model has clinical value for guiding treatment. This study can also provide good evidence for future meta-analyses.

## 5 Conclusions

In conclusion, the risk-assessment nomogram constructed in this study integrates available clinical and therapeutic characteristics. Clinicians can use more comprehensive information to aid in clinical decision-making for patients who receive COS treatment. Future analyses need to include additional possible influencing factors to improve the discriminative performance of the model, and prospective research and external data validation are needed.

## Data Availability

The data analyzed in this study is subject to the following licenses/restrictions: The data utilized and analyzed in the current study is accessible from the corresponding author upon reasonable request. Requests to access these datasets should be directed to HK, luckjuanjuan819@126.com.
