# Supplementary material for: Development and validation of a prediction model for premature progesterone elevation during early-follicular phase long-acting GnRH-a long protocol
Source: Front Endocrinol (Lausanne). 2026 Jul 22;17:1847048. doi: 10.3389/fendo.2026.1847048 (PMC13437324; doi:10.3389/fendo.2026.1847048)
Supplement: Supplementary file 2 [file Table2.docx]

**Supplementary Table 2 Results of multivariate logistic regression for training cohort of sensitivity analysis**

| **Characteristic** | **OR** | **95% CI** | **p-value** |
| --- | --- | --- | --- |
| Age (year) | 1.01 | 1.00, 1.03 | 0.161 |
| BMI (kg/m2) | 0.94 | 0.92, 0.96 | <0.001 |
| Basal FSH (mIU/ml) | 0.91 | 0.88, 0.95 | <0.001 |
| Basal P (ng/ml) | 1.43 | 0.97, 2.11 | 0.074 |
| AMH (ng/ml) | 0.95 | 0.92, 0.98 | <0.001 |
| AFC | 0.96 | 0.94, 0.97 | <0.001 |
| Total duration days of exogenous Gn (day) | 1.33 | 1.27, 1.40 | <0.001 |
| Total dosage of exogenous HMG (IU) | 1.00 | 1.00, 1.00 | <0.001 |
| FSH on the 6th day of exogenous Gn (mIU/ml) | 1.03 | 1.01, 1.06 | 0.004 |
| P on the 6th day of exogenous Gn (ng/ml) | 4.46 | 2.33, 8.54 | <0.001 |
